# Facilitators and Barriers for Private Health Sector Engagement for TB Care in India: A Systematic Review and Meta-Synthesis of Qualitative Research

**DOI:** 10.9745/GHSP-D-24-00034

**Published:** 2024-08-27

**Authors:** Rakesh PS, Mohd Shannawaz, Manu E. Mathew, Kuldeep Singh Sachdeva

**Affiliations:** a Amity Institute of Public Health & Hospital Administration, Amity University, Noida, India.; b The Union South East Asia Office, New Delhi, India.

## Abstract

**Introduction:**

Private sector engagement is recognized as one of the most critical interventions to achieve the End TB goals in India. We conducted a systematic review and a meta-synthesis of qualitative studies to identify the barriers and facilitators for private sector engagement in TB care in India.

**Methods:**

A systematic search in electronic databases was done. We assessed the methodological limitations of individual studies, synthesized the evidence using thematic analysis, and assessed our confidence in each finding.

**Results:**

Of the 19 eligible articles included for the qualitative synthesis, 31.5% (6/19) were conducted in northern states of India. Included studies had details from 31 focus group discussions and 303 in-depth interviews conducted among various stakeholders. The synthesis revealed that barriers to engaging the private sector were lack of coordination mechanisms, lack of the National TB Elimination Program (NTEP) staff capacity to deal with the private sector, lack of private practitioners’ knowledge on various programmatic aspects, and perceived complexity of the data exchange mechanism. The private sector felt that NTEP was not sensitive to the patient’s confidentiality and demanded too much patient data. The private sector considered nonfinancial incentives like recognition, feedback, involving them in planning, and giving them equal status in partnership as powerful enablers for their engagement in TB care.

**Conclusion:**

Factors related to the context in which the engagement occurs, the architecture of the engagement, and interaction among the actors contribute to barriers to engaging the private sector for TB care in India. Strengthening policies to protect patient confidentiality, using behavior change communication to NTEP program managers, providing managerial and soft-skill training to NTEP staff, promoting nonfinancial incentives to private providers, establishing a coordination mechanism between the sectors, and simplifying the data exchange mechanisms need to be done to further strengthen the private-sector engagement.

## Abstract (precis)

To strengthen the private sector engagement in TB care in India, several strategies should be considered, such as promoting nonfinancial incentives to private providers, establishing a coordination mechanism between public and private sectors, and simplifying data exchange mechanisms.

## Teaser

**Key Findings:**

- Despite efforts by the National TB Elimination Program (NTEP) to engage the private sector in providing timely, high-quality TB care and services, private-sector engagement in India remains suboptimal.
- A meta-synthesis of qualitative findings from 19 studies included in the review found that although private-sector providers were committed to contributing to the fight against TB, they expressed several concerns that posed barriers to partnering with NTEP.
- Barriers to private-sector engagement included for-profit private-sector providers expressing concerns about losing business to NTEP, lack of coordination between sectors, concerns about patient confidentiality and privacy, and negative attitudes and lack of capacity of NTEP staff to deal with private-sector providers.
- Enablers for private-sector engagement included nonfinancial incentives like recognition, feedback, involvement in planning, and consideration as equal status in the public-private partnership.

**Key Implications:**

- Policymakers should address barriers to private-sector engagement in TB care and develop a comprehensive engagement strategy and uniform understanding among all stakeholders.
- District- and state-level program managers need to clearly understand the vision of private-sector engagement for TB care and support the private sector to achieve the same as equal partners in the fight against TB.

## INTRODUCTION

With an estimated 2.8 million new TB cases occurring annually, India contributes to 26% of the global burden of incident TB cases.^1^ In India, at least half of the people with TB symptoms seek care from the private sector.^2^^,^^3^ However, there are concerns about the suboptimal quality of TB care in the private sector due to the use of incorrect diagnostic and nonstandardized treatment protocols, lack of systems for ensuring treatment adherence, lack of patient support and contact investigations leading to delayed diagnoses, and high rate of unsuccessful treatment outcomes eventually increasing the risk of drug resistance.^4^^–^^6^

Nearly 2 decades ago, India’s TB program realized the need to engage the private sector and initiated several initiatives for public-private mix. To engage with the private sector, the National TB Elimination Program (NTEP), previously known as the Revised National TB Control Program, has used a variety of approaches, including education, regulation, provision of free services, incentives, and partnership schemes. We recently published a detailed review of these efforts.^7^ The National Strategic Plan (NSP) for TB Elimination in India (2017–2025) established strategies to ensure that patients reaching the private sector receive timely and quality-assured diagnosis and treatment, protection from high out-of-pocket expenditure, other public health services (e.g., management of comorbidities), contact investigation and disease prevention, counseling, adherence support and monitoring, nutritional support, and outcome reporting.^8^ Despite the NTEP’s efforts, private-sector engagement remains suboptimal.^7^^–^^9^

Qualitative studies offer a good description of a phenomenon that encompasses all the complexity of a phenomenon, context, or behavior. Meta-synthesis can advance current knowledge by combining qualitative insights from many studies on the topic of interest. Meta-synthesis has been suggested as an appropriate method to closely inquire into the phenomena from the perspectives of those who are affected by the phenomena as well as the interpretations of the investigators.^10^ This study attempts to answer the question: what does qualitative research tell us about the barriers and facilitators for engaging the private health service delivery sector for TB care in India? Such insightswill be helpful for policymakers and program managers to further strengthen the partnership with the private sector.

Insights on barriers and facilitators of private-sector engagement will help policymakers and program managers further strengthen the partnership with the private sector.

## METHODS

### Scope and Definitions

We used the operational definition of the private sector provided by the World Health Organization, which defined it as “the individuals and organizations that are neither owned nor directly controlled by governments and are involved in provision of health services.”^11^ We focused on for-profit private health service delivery providers, including both formal and informal providers, because they are more numerous and difficult to engage. We defined informal health care providers as those who have not received any formally recognized training with a defined curriculum from an institution, are not typically registered with any government regulatory body, collect payment from patients served, and operate outside of the purview of government or other institutions.

We used the definition of private sector engagement as “the meaningful inclusion of private providers for service delivery in mixed health systems,” as defined by the World Health Organization Advisory Group on the Governance of the Private Sector for Universal Health Coverage.^12^ The definition is broad to capture all modalities for engaging the private sector, from informal collaborations to more formalized partnerships.

### Selection Criteria

We included primary studies that used both qualitative methods for data collection (focus group discussions (FGDs), in-depth interviews (IDIs), key-informant interviews (KIIs), and field notes) and qualitative methods for data analysis (e.g., thematic analysis and grounded theory). We included mixed-method studies where we could extract the data collected and analyze it using qualitative methods. We included studies that described the perspectives of private providers, program managers, intermediary agencies, or patients.

We included only those studies focused on “for-profit” health service delivery providers, including both formal and informal providers. We included only health service delivery providers rather than manufacturers or distributors of medical equipment, technologies, consumables, or drugs.

We excluded publications that did not report on primary research. We also excluded studies that were not peer reviewed, not in English, and done outside India. All published studies between January 1, 2000, and August 30, 2023 were included.

### Search Methods

We initially conducted a scoping search to become familiar with the literature and gain insights to identify keywords and medical subject headings. Search terms and strategies are described in Table 1. PS conducted a systematic search in Medline (OVID) on September 4, 2023. Additional searches were done in Embase (OVID), Scopus, and Web of Science. Using citation chaining, we searched the reference lists of selected articles to find additional studies.

**TABLE 1.** Search Terms and Strategies Used to Search Electronic Databases

1	MeSH: Private Hospitals OR Private Sector OR Private Enterprise OR Public Private Partnership OR Public-Private Cooperation OR Public-Private Partnership OR Public-Private Partnerships OR Public-Private Sector Cooperation OR Private Facilities
2	MeSH: Tuberculosis Ti/Ab: (Tuberculosis OR Tuberculoses OR TB)
3	MeSH: India
4	Ti/Ab: (Facilitat* OR Promot* OR Barrier* OR Success OR succeeded OR Issue* OR Factor* OR Concern* OR Hurdle* OR Obstacle* OR Achieve* OR Accomplish* OR Enable* OR learning* OR challenge* OR contrain* OR Enhanc* OR Influenc* OR problem* OR Interfer*)
Search	1 AND 2 AND 3 AND 4

### Selection of Studies

Initial screening was done by PS. After removing duplicates, 2 review authors (PS, MS) assessed the titles and abstracts independently to evaluate eligibility. A screening tool with prespecified study inclusion/exclusion criteria was used to avoid any subjectivity, and reasons for exclusion were clearly documented. When unsure at the screening stage, studies were included in full-text screening. We then retrieved the full texts of all articles identified as potentially relevant and assessed these articles independently. We resolved any disagreements by involving a third review author (MM) and through discussions.

### Data Extraction

PS developed and piloted a data extraction form on 3 studies. The completed forms were then reviewed by a second review author (MM) for accuracy and completeness. Two review authors (PS, MS) individually performed data extraction using the finalized data extraction form. We extracted the following information from each study: year of data collection, year of publication, study setting, health care provider participated, sample size, study design, and data collection methods. We contacted 2 of the authors of the studies by email to clarify the number of interviews with each type of health care provider in their study.

### Quality Assessment

PS and MS independently assessed each study for methodological limitations, and we resolved disagreements through discussion between authors. We used the CASP (Critical Skills Appraisal Programme) tool for assessing the methodological limitations.^13^ We did not exclude studies based on our assessment of methodological limitations. We used the information about methodological limitations to assess our confidence in the review findings.

### Data Synthesis

Authors familiarized themselves with the qualitative data reported and used initial inductive coding (PS, MM) that was subsequently refined based on discussions (PS, MS, MM, KS). Codes were grouped into analytical categories and subcategories from which overarching themes and subthemes were generated through an iterative process (PS, MS) and checked for consistency and validity (PS, MS, MM, KS). We rearranged the data according to relationships, mapped and interpreted the nature of reviewed concepts, and looked for how the themes addressed the review question. During all stages of data synthesis, regular meetings of the review team facilitated critical discussion and interrogation of the data. Disagreements, if any, were discussed and resolved between the reviewers. An expert (SB) in public-private mix, who was not part of the study team, reviewed the synthesized findings, which facilitated trustworthiness, coherence, and relevance of the findings.

### Assessing Confidence in the Review Findings

Two review authors (PS, MM) independently used the GRADE-CERQual (Confidence in the Evidence from Reviews of Qualitative research) approach to assess our confidence in each finding.^14^ GRADE-CERQual assesses confidence in the evidence based on 4 key components: (1) methodological limitations of included studies, (2) coherence of review finding, (3) adequacy of data contributing to a review finding, and (4) relevance of included studies to the review question. After assessing each of the 4 components, we judged the overall confidence in the evidence supporting each review finding as high, moderate, low, or very low. All findings started as high confidence and were then graded down if there were important concerns regarding any of the GRADE-CERQual components.

## RESULTS

The Figure shows the PRISMA flow diagram of our search results and the process of screening and selecting studies for inclusion.^15^ We screened 276 titles and abstracts, shortlisted 42 articles for full-text review, and included 19 articles for the qualitative synthesis. The characteristics of the studies included are provided in Supplement 1.^16^^–^^35^

**FIGURE:**
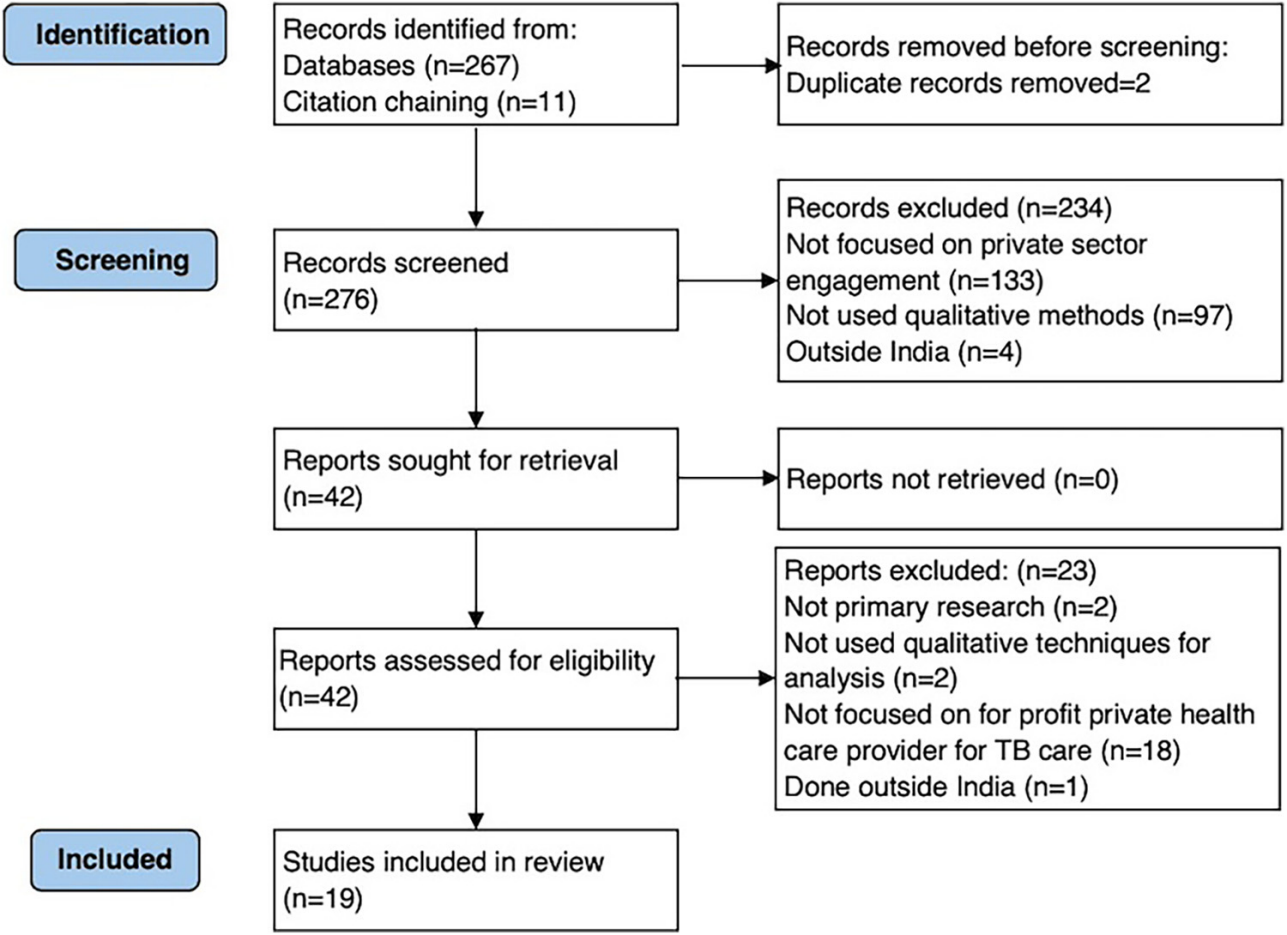
PRISMA Flowchart Indicating the Results of Literature Search

### Description of Studies Included

Of the 19 studies included, 13 (68.4%) studies were published within the last 5 years (2019–2023).^16^^–^^28^

### Study Settings

Six (31.5%) studies were done in Karnataka state,^20^^,^^22^^,^^23^^,^^27^^,^^29^^,^^31^ 4 (21%) in Kerala,^19^^,^^24^^,^^28^^,^^31^ 2 (10.5%) each from Maharashtra^33^^,^^34^ and Delhi,^16^^,^^30^ and 1 each from Himachal Pradesh,^17^ Telangana,^21^ Gujarat,^25^ Kolkata,^18^ and Bihar.^26^

### Topics of Interest

Ten (52.6%) studies focused on enablers and barriers for TB notification from the private sector,^16^^–^^18^^,^^22^^,^^23^^,^^25^^,^^27^^,^^30^^,^^32^^,^^33^ 2 (10.5%) focused on the complex relationship among actors in partnership,^29^^,^^34^ 2 (10.5%) on experiences of ongoing private-sector engagement activities,^24^^,^^31^ 2 (10.5%) on chemist’s engagement,^19^^,^^26^ and 1 each on perception of stakeholders on policy,^20^ enablers and barriers for involving private practitioners in signed partnership schemes,^28^ and involvement of informal health care providers.^21^

### Designs and Methods

Eight (42.1%) studies were mixed methods design^17^^,^^18^^,^^21^^,^^22^^,^^25^^,^^27^^,^^32^^,^^33^ and 2 (10.5%) were done as part of an evaluation of interventions.^24^^,^^26^ Ten (52.6%) studies used IDIs alone, 3 (15.75) used FGDs alone, and 6 (31.6%) used a combination of various techniques. Overall, 16 (84.2%) studies used IDIs, and 8 (42.1%) studies used FGDs. Three (15.8%) studies were done by the same author (Solomon et al.) in the same setting (a TB Unit area in Karnataka) with different objectives using a combination of ethnography, participant observations, IDIs, and FGDs.^20^^,^^29^^,^^31^

### Study Participants

There were a total of 31 FGDs (15 with private doctors including specialists, 9 with chemists, 4 with NTEP staff, 1 each with nurses, ayurveda, yunani, siddha, homeopathy [AYUSH] providers, and informal health care providers) and 303 IDIs (88 with NTEP program personnel, 86 modern medicine general practitioners, 45 modern medicine specialists, 27 chemists/chemist shop owners, 16 AYUSH practitioners, 8 people affected with TB, 7 modern medicine doctor’s association leaders, 7 drug enforcement officers, 5 nurses, 4 hospital administrators, 4 laboratories, 3 chemist association leaders, 2 developmental partners, and 1 informal health care provider). Perspectives of modern medicine practitioners, including specialists, were captured in 14 (73.7%) studies, NTEP program personnel in 11 (57.8%) studies, AYUSH practitioners in 4 (21%) studies, informal health care providers in 2 (10.5%) studies, and hospital administrators and patients in 1 study each.

Twelve (63.1%) studies did not provide details on participants’ gender,^18^^,^^20^^,^^21^^,^^23^^.^^24^^,^^26^^,^^28^^–^^33^ while in the remaining 7 studies, the percentage of female participants ranged from 7% to 81%.

### Methodological Limitations of the Studies

The details of methodological limitations are provided in Supplement 2.

### Review Findings

Overarching themes, subthemes, and codes emerged during inductive coding (Table 2). We classified the characteristics of private health service providers as (1) corporate/private hospitals, (2) formal qualified modern medicine practitioners/nursing homes, (3) AYUSH providers, (4) chemists, (5) informal health care providers, and (6) private laboratories. We also captured the perspectives of (1) NTEP, (2) private providers, (3) intermediaries, and (4) patients separately.

**TABLE 2.** Summary of Themes, Subthemes, and Emergent Codes for Meta-Synthesis of Qualitative Data on Private Sector Engagement in TB Care in India

**Overarching Themes**	**Subthemes**	**Emergent Codes**
Context in which the engagement occurs	Political and economic factors	Stakeholder interests, reasons for poor interest, social responsibility, market forces
	Internal environment of the organizations	NTEP and private sector: value system, vision and goals, organizational structure, culture of organization, stewardship, motivation
	Sociocultural factors	Stigma, confidentiality, patient preferences
Factors that define the architecture of the engagement and its implementation	Strategies for engagement	Policy and dialogue: acceptance Regulatory approach: mandatory TB notification, schedule H1 regulation, social regulations–enablers and concerns Incentives: financial and nonfinancial, enablers and concerns Information exchange: enablers and concerns Public provision of services (drugs, diagnostics, training, public health actions): acceptance, concerns Financing: partnership schemes, strategic purchase, insurance, subsidy
	Mode of engagement	Intermediary agencies, private sector led initiatives, memorandum of understanding, contracts
	Resources for engagement	Human resources: adequacy, workload Other resources: finance, drugs, diagnostics, technological resource, informational resource
	Translation of policies to practice	Knowledge gaps, policy translation to practice, implementation of strategies, procedural hurdles and delays, enablers and barriers
Factors related to the actors implicated in the engagement	Relationship dynamics	Trust, mutual understanding, prior experiences, accountability, positionality
	Capacities to engage	Managerial and technical capacities to engage
	Interaction of actors	Communication, interaction, flexibility, coordination of process, mutuality

Abbreviations: NTEP, National TB Elimination Program.

Findings from the qualitative synthesis are summarized in Table 3. Details of evidence profiles for GRADE CERQal for assessing the confidence level in each finding are provided in Supplement 3.

**TABLE 3.** Summary of Review Findings From the Meta-Synthesis of Qualitative Studies

**Review Finding Summary**	**Studies Contributing to Review Finding**	**GRADE-CERQual Assessment of Confidence in Evidence**	**Explanation of CERQual Assessment**
1. Private health service delivery sector considered it their professional responsibility to provide quality TB services to all their clients and were committed to contribute to society in fighting TB.	Solomon et al. (2016, 2018),^29^^,^^31^ Nair et al.^28^ Daftary et al.,^26^ Rakesh et al.,^24^ Bharadwaj et al.^17^	Moderate	No or minor concerns regarding coherence, moderate concern regarding relevance and adequacy, and minor concern regarding methodological limitations.
2. While engaging with NTEP, formal health care providers in private sector had concerns of “losing their business,” fear of scrutiny of diagnosis and loss of their “autonomy” to diagnose and treat.	Anand et al.,^22^ Solomon et al. (2016, 2018),^29^^,^^31^ Sairu. et al.,^32^ Nair et al.,^28^ Yeole et al.,^33^ Ghatage et al.^23^	High	No or minor concerns regarding coherence, relevance, adequacy, and methodological limitations.
3. Patients and the private sector doctors had a concern that NTEP is not sensitive to the patient’s confidentiality and privacy.	Anand et al.,^22^ Rashmi et al.,^16^ Sairu et al.,^32^ Nair et al.,^28^ Mahasweta et al.,^30^ Yeole et al.,^33^ Ghatage et al.,^23^ Shukla et al.,^18^ Rupani et al.^24^	High	No or minor concerns regarding coherence, relevance, adequacy, and methodological limitations.
4. Establishing a single window system inside a private hospital could be a facilitator for improving quality of services to clients with TB.	Archana et al.,^27^ Rakesh et al.,^24^ Yeole et al.,^33^ Sairu et al.^32^	Moderate	No or minor concerns regarding coherence, moderate concern regarding relevance and adequacy, and minor concern regarding methodological limitations.
5. Engaging hospital administrators could be a facilitator for engaging private hospitals.	Nair et al.,^28^ Rakesh et al.,^24^ Archana et al.^27^	Low	No or minor concerns regarding coherence, moderate concern regarding relevance, serious concern regarding adequacy, and minor concern regarding methodological limitations.
6. Judicious use of Schedule H1 drug regulation for anti-TB drugs could enable private sector engagement through identification of right providers.	Rakesh et al.^19^	Very low	No or minor concerns regarding coherence, moderate concern regarding relevance, serious concern regarding adequacy, and minor concern regarding methodological limitations.
7. Private hospitals’ and modern medicine practitioners’ motivation to engage was not driven by financial incentives provided by NTEP.	Solomon et al. (2016, 2018),^29^^,^^31^ Nair et al.,^28^ Rakesh et al.^24^	Low	Moderate concern regarding coherence, minor concern regarding relevance, moderate concern regarding adequacy, and minor concern regarding methodological limitations.
8. Financial incentive might be useful for engaging informal health care providers and chemists, if provided timely.	Daftary et al.,^26^ Solomon et al. (2018),^29^ Kelamane et al.^21^	Low	No or minor concerns regarding coherence, moderate concern regarding relevance, serious concern regarding adequacy, and minor concern regarding methodological limitations.
9. Delay in disbursement of committed funds was very common and it could lead to loss of trust among partners.	Nair et al.,^28^ Solomon et al.^20^	Low	No or minor concerns regarding coherence, moderate concern regarding relevance, serious concern regarding adequacy, and minor concern regarding methodological limitations.
10. Private sector considered non-financial incentives like recognition, feedback, involving them in planning and review and giving them equal status in partnership as powerful enablers for their engagement for TB care.	Shukla et al.,^18^ Nair et al.,^28^ Solomon et al.,^31^ Bharadwaj et al.,^17^ Rakesh et al.^24^	High	No or minor concerns regarding coherence, relevance, adequacy, and methodological limitations.
11. Private sector felt that NTEP is demanding “too” much of patient wise data and the system for information exchange needs to be simplified.	Anand at al,^22^ Rashmi et al.,^16^ Sairu et al.,^32^ Mahasweta et al.,^30^ Daftary et al.,^26^ Yeole et al.,^33^ Ghatage et al.,^23^ Shukla et al.,^19^ Bharadwaj et al.,^17^ Rupani et al.^24^	High	No or minor concerns regarding coherence, relevance, adequacy, and methodological limitations.
12. Private-led initiatives to improve quality of TB care such as STEPS had wider acceptance among all stakeholders.	Rakesh et al.^24^	Very low	No or minor concerns regarding coherence, moderate concern regarding relevance, serious concern regarding adequacy, and minor concern regarding methodological limitations.
13. Lack of coordination mechanisms between public and private sector was a major barrier for private sector engagement.	Anand et al.,^22^ Sairu et al.,^32^ Yeloe et al.,^33^ Rakesh et al.,^24^ Shukla et al.^19^	High	No or minor concerns regarding coherence, relevance, adequacy and methodological limitations.
14. NTEP staff lacked capacity to deal with private sector and require technical, managerial and soft skill training.	Anand et al.,^22^ Solomon et al.,^29^ Karina et al.,^34^ Nair et al.,^28^ Mahasweta et al.,^30^ Yeole et al.^33^	High	No or minor concerns regarding coherence, relevance, adequacy, and methodological limitations
15. Lack of uniform understanding regarding private sector engagement among NTEP district officials and field staff hindered the sustainable engagement of private sector for TB care.	Solomon et al. (2018, 2021),^29^^,^^31^ Karina et al.,^34^ Rakesh et al.^24^	Very low	No or minor concerns regarding coherence, moderate concern regarding relevance, serious concern regarding adequacy, and minor concern regarding methodological limitations.
16. There was lack of knowledge about the relevant programmatic aspects (e.g., Ni-kshay, misconceptions about notification) among private sector providers.	Yeole et al.,^33^ Bharadwaj et al.,^17^ Shukla et al.,^18^ Archana et al.,^27^ Sairu et al.,^32^ Anand et al.,^22^ Rupani et al.^25^	High	No or minor concerns regarding coherence, relevance, adequacy and methodological limitations.
17. “Authoritarian” approach of NTEP district-level officials led to inequality in public-private partnerships and was perceived by the private sector as a major barrier for engagement.	Solomon et al. (2016, 2018, 2021),^20^^,^^29^^,^^31^ Nair et al.,^28^ Sairu et al.^32^	Moderate	No or minor concerns regarding coherence, moderate concern regarding relevance and adequacy and minor concern regarding methodological limitations
18. Sustained interaction of NTEP with private sector was an enabler for successful engagement of private sector.	Anand et al.,^22^ Solomon et al. 2021.^20^	Very low	No or minor concerns regarding coherence, moderate concern regarding relevance, serious concern regarding adequacy, and minor concern regarding methodological limitations.

### Theme 1: Context in Which Engagement Occurs

#### Finding 1: Contribution to TB Care

Findings from various studies pointed out that private health care providers considered it their professional responsibility to provide quality TB services to all their clients and that many private providers felt a sense of pride while contributing to TB care.^17^^,^^24^^,^^26^^,^^28^^,^^29^^,^^31^

*I feel good that I am being able to serve my society. People are benefitting. We are able to provide care and people are getting better.* ^26^

*Private sector is always willing to help Government in dealing with social issues like TB.* ^24^

#### Finding 2: Concerns of Loss of Clients and Autonomy

Though the private sector was committed to contributing to society by fighting TB, they had many concerns while partnering with NTEP.^22^^,^^23^^,^^28^^,^^29^^,^^31^^–^^33^ Formal health care providers had concerns about “losing their business” while engaging with NTEP, as, at times, NTEP “pulls away” their clients. They also worried that the “government” would scrutinize their diagnosis. They were also afraid that their “autonomy” to diagnose and treat would be lost if there was too much engagement.

Private-sector providers were afraid that they would lose autonomy to diagnose and treat clients if there was too much engagement with NTEP.

#### Finding 3: Concerns About Patient Confidentiality and Privacy

Patient confidentiality was a concern, which most of the private practitioners raised regarding the sharing of patient information with NTEP. Patients and private sector doctors were concerned that NTEP was not sensitive to patients’ confidentiality and privacy.^16^^,^^18^^,^^22^^–^^24^^,^^28^^,^^30^^,^^32^^,^^33^

*If we report (to NTEP), the patient might be followed up and sometimes patient feel that their privacy is being breached.* ^16^

*… Her father begged not to send his daughter to the government or report her diagnosis to anyone. I knew the family for a long time. I thought I will ensure treatment and its completion. I didn’t notify as I had to respect his wish.* ^32^

*Patient may not like to disclose their TB status.* ^33^

### Theme 2: Factors That Define the Architecture of Engagement and Its Implementation

#### Finding 4: A Single-Window System to Improve TB Care

In private hospitals, there is no system for coordinating the response and providing complete TB care, including treatment adherence monitoring and support. Patients are treated in various departments, and not all departments are aware of the entire spectrum of TB services. Where attempts were made to create a “single-window” system within the hospital, there was proven success.^24^^,^^27^^,^^32^^,^^33^ Such success was seen in the System for TB Elimination in Private Sector (STEPS) model in Kerala and in a private tertiary care hospital in Karnataka.^24^^,^^27^ In STEPS, a nodal person within a hospital designated by the hospital management, typically a staff nurse, acts as a single point of contact for all TB-related services, including linkage to diagnostic and treatment services, notification, patient linkage with social welfare, contact investigations, TB preventive therapy, treatment adherence support, coordination with NTEP, and assistance to clients to navigate the system.

#### Finding 5: Engagement of Hospital Administrators

Nair et al. reported that failure to convince hospital management was a critical barrier to engaging the private sector.^28^ Stakeholders of the STEPS initiative in Kerala reported that the biggest facilitator for private hospital engagement was support from hospital administration.^24^

*We failed in convincing hospital managements. Doctors can’t overrule hospital managements.* ^28^

#### Finding 6: Judicious Use of Schedule H1 Drug Regulation

To monitor the indiscriminate use of certain antibiotics and prevent the emerging threat of resistance to antimicrobial agents, in 2014, the Government of India established Schedule H1 regulation, which mandated that anti-TB drugs could be sold only if there was a valid prescription by a modern medicine practitioner. The chemist also needed to maintain a separate Schedule H1 register that includes the patient’s identity, prescribing doctor’s contact information, drug name and dispensed quantity, and date.

Experiences from Kerala documented that schedule H1 surveillance could help in identifying providers who had not notified TB and could prioritize them for engagement.^19^ However, perspectives regarding regulations could be found only in a single but well-conducted study.

*Based on Schedule H1 data, I used to write friendly letters to doctors who did not notify TB offering them support for notifications. Now they inform all TB cases the moment they diagnose.* ^19^

#### Finding 7: Motivation to Engage Not Driven by Financial Incentives

Private-sector modern medicine doctors and hospital management staff stated that they were not keen to receive financial incentives from NTEP for information exchange. Many studies reported that private doctors and hospitals were least interested or motivated by the financial incentives given by NTEP.^24^^,^^28^^,^^29^^,^^31^ Respondents in some studies also expressed concerns about the misuse of incentive-driven notification for monetary gain by any sector or intermediary agencies.^22^ In a study from Kolkota, there was a casual mention about the desire for financial incentives by private sector doctors; however, the argument was not convincing due to a lack of details in the study.^18^

*As a private doctor, I am not interested in Rs 250. What I need is the freedom to prescribe for my patients.* ^29^

*Money is not everything and private sector is willing to collaborate even without financial assistance.* ^28^

*We are even willing to forgo our profits for TB patients - a private hospital administrator of a 100 bedded hospital.* ^24^

#### Finding 8: Financial Incentives to Engage Informal Health Care Providers and Chemists

A few studies observed that financial incentives might be useful for engaging informal health care providers and chemists.^21^^,^^26^^,^^29^ Studies also reported that they expected timely payments and any delay in payments would be counterproductive.

*… Financial incentives were secondary for them, field observations showed that financial incentives were the crucial factor in motivating unqualified practitioners to be involved when compared to those individuals with qualifications.* ^29^

*… Though incentive is not of great attraction to them [informal providers] … at times … they complain about not receiving it! they expect it to happen immediately … how is it possible?* ^21^

#### Finding 9: Delay in Disbursement of Committed Funds

Some studies reported that lack of disbursement of committed funds by NTEP led to loss of trust among the partners.^20^^,^^28^ The program managers were not confident about their ability to release the funds in a timely manner to partners.

*Another reason why the programme managers are not so willing to enter into formal contractual arrangements is the lack of confidence in their own ability to release funds on time for supporting such initiatives.* ^28^

#### Finding 10: Nonfinancial Incentives Are Powerful Enablers for Engagement

Generally, private providers preferred more nonfinancial forms of incentives, such as receiving recognition, getting involved in planning, receiving timely feedback and knowledge updates, and considering them as equal partners. There was good coherence among studies in this regard.^17^^,^^18^^,^^24^^,^^28^^,^^31^

*We were never invited for a meeting nor involved in planning process. What we have is only 1-time sensitization.* ^28^

*Nonfinancial incentives like recognition, trainings, involving them in planning and review meetings and giving them equal status is more than enough for private sector to engage in TB control.* ^28^

*… Government should keep motivating the private sector.* ^19^

#### Finding 11: Need for Information Exchange Simplification

Private-sector providers felt that NTEP demanded too much patient information from them.^16^^,^^17^^,^^19^^,^^22^^–^^24^^,^^26^^,^^30^^,^^32^^,^^33^ They were not too keen to invest that much time for information exchange. Doctors were generally too busy in clinical management and were neither interested nor fully aware of the process of information exchange through Ni-kshay.

Private-sector providers were not too keen to invest a lot of time for information exchange with NTEP.

*Reporting should be made hassle free and easy so that it does not too much time and extra manpower is not required.* ^30^

*That form (TB notification form) has too many details. It should be reframed.* ^23^

*… First registering then uploading patient data and updating-it is complex.* ^19^

#### Finding 12: Private-Led Initiatives Had Wider Stakeholder Acceptance

STEPS is a private-sector-led initiative to address gaps in the quality of TB care in the private sector. STEPS was envisioned as an equal partnership between the public and private sectors for the benefit of society, where both sectors are held accountable for improving the quality of TB care. It has been piloted in Kerala state with the involvement of 340 private hospitals. An evaluation of STEPS consisting of 33 qualitative interviews with different stakeholders confirmed that it was an acceptable model for all stakeholders, including NTEP, private providers, and patients.^24^

*STEPS is one of the best initiatives that I have seen in my overall career of 24 years in NTEP. We had so many issues with PPP. No doctor will hear us and were willing to see us previously. Now the communication is very smooth as we have contact person (STEPS Leads) in every hospital. We have a WhatsApp group also with all STEPS Leads. It has made our life so simple.* ^24^

#### Finding 13: Lack of Coordination Mechanisms Between Public and Private Sector

Private sector providers felt that there was no platform for dialogue between the public and private sectors nor a well-coordinated mechanism for smooth communication. Many studies reported that lack of a coordination mechanism hindered engagement.^19^^,^^22^^,^^32^^,^^33^ Experiences from STEPS in Kerala showed the benefits of how a well-coordinated mechanism through private hospital consortiums facilitated the partnership.^24^

*… Continuous dialogue should be there between government doctors and PPs.* ^33^

### Theme 3: Factors Related to the Actors Implicated in the Engagement

#### Finding 14: Need for NTEP Staff Capacity-Building

All the studies that captured the NTEP staff’s perspectives clearly concluded that the NTEP staff who visited private hospitals and private-sector doctors lacked the capacity to deal with the private sector.^22^^,^^28^^–^^30^^,^^33^^,^^34^ These staff lacked technical competency to talk to private-sector doctors and often were unable to answer all their questions.

NTEP staff reported that they lacked technical competency and capacity to answer private-sector doctors’ questions.

*I think an MBBS doctor only should approach them, then they will speak with respect. If we go, they say go, we are very busy now; they do not give us respect.* ^29^

*It is very difficult for us even to meet a doctor in a private hospital. They will be busy seeing their patients. We need to wait for long time like a medical representative.* ^28^

*Someone needs to take the responsibility to speak to private doctors, rest we can handle. People with higher position with higher responsibility can take initiative.* ^30^

#### Finding 15: Misunderstanding Regarding Private-Sector Engagement

Many studies documented that NTEP program managers and staff misunderstood private sector engagement as a means to achieve the notification and outcome “targets.”^29^^,^^30^^,^^34^ There was a general failure to comprehend the scope of the partnership. There were documented conflicting views of program managers regarding “private sector engagement.”^29^^,^^34^

*To involve means increasing the cases…what doctors [Higher Officers] tell us is that at each centre there has to be minimum of 10-15 sputum positive cases… from “Out Patient” we only get 2-3 cases. So to get more cases we need to approach the PPs.* ^29^

*My experiences with government were always been bad. Every time the person changes, their response also changes.* ^24^

#### Finding 16: Lack of Private-Sector Provider Knowledge About Certain Program Aspects

Many studies documented that private providers generally lacked understanding about relevant programmatic aspects (e.g., notifications through Ni-kshay) and had a lot of misconceptions about TB notification.^17^^,^^18^^,^^22^^,^^25^^,^^27^^,^^32^^,^^33^ Private providers also found it difficult to attend the training sessions organized by NTEP or intermediary agencies due to their busy working schedules, and being away from duty also meant “loss of livelihood” for them.

#### Finding 17: Inequality in Public-Private Partnerships

A few studies documented that district-level NTEP officials followed an “authoritarian,” “dominating,” “overpowering,” and even a “hostile” approach when dealing with the private sector.^20^^,^^28^^,^^29^^,^^31^^,^^32^ Private practitioners felt undervalued in the entire process and felt that the entire private-sector engagement was devised from the NTEP’s perspective without considering their views and concerns. Partners working as intermediaries also felt that NTEP considered them inferior, leading to inequality in partnerships.

*Approach to private by government is very bad. They do not give us respect.* ^28^

*I do not like the step motherly attitude of the government to private.* ^32^

#### Finding 18: Sustained Interaction Enabled Successful Engagement

Wherever the NTEP staff visited private practitioners frequently, there was successful engagement. Experiences from Pune and Karnataka revealed that repeated visits of NTEP staff to private practitioners ensured a trusting relationship.^20^^,^^22^ Sustained interactions were considered an enabler for successful engagement of the private sector.

## DISCUSSION

The current study aims to synthesize evidence from qualitative studies to identify barriers and facilitators for private-sector engagement in TB care in India. To our understanding, this is the first attempt to synthesize evidence from qualitative studies to identify facilitators and barriers to private-sector engagement in TB care.

To summarize, the current review focuses on facilitators and barriers to engaging the private sector for TB care in India and summarizes major findings through a meta-synthesis of qualitative studies. Although private sector providers were committed to contributing to fighting TB, several studies reported they had concerns about losing clients, fear of scrutiny, losing autonomy to diagnose and treat clients, and concerns about patient confidentiality and privacy. Other major barriers to engaging the private sector included NTEP program managers using an authoritarian approach in interactions, a lack of coordination mechanisms between the public and private sectors, and NTEP staff lacking the capacity to deal with the private sector. There is also a lack of knowledge about the relevant programmatic aspects among private sector providers. The private sector considers nonfinancial incentives like recognition, feedback, involvement in planning and review, and equal status in partnership as powerful enablers for their engagement in TB care. Engaging hospital administrators, establishing a single window system inside a private hospital, and sustained interaction between sectors could be other major facilitators for improving the quality of TB care and services.

The review has increased our understanding of what helps or hinders private-sector engagement in TB care in India. We believe that the findings will allow managers and policymakers to see clearly the strategies that need to be designed or modified to further strengthen public-private partnerships.

Some of the findings reported in the current study reiterate findings from similar reviews focusing on private-sector engagement. Similar to our conclusion, a systematic review on models of public-private sector diagnostic and referral services for TB/HIV coinfected patients also identified a lack of coordination as a challenge for partnerships.^35^ Similar to our review findings, another systematic review that explored the role of private practitioners in disease surveillance activities identified the main barriers to private-sector participation as inadequate knowledge and misperceptions that influence their practices, complicated reporting mechanisms, and unsatisfactory attitudes of the government surveillance program managers toward the private sector.^36^

It is recommended to have a clear strategy for private-sector engagement that will help to avoid confusion among stakeholders. Behavior change strategies need to be devised to encourage a uniform outlook for state, district, and subdistrict NTEP health officials regarding private sector engagement and enable them to treat the private sector as equal partners. The capacities of peripheral staff, such as public-private mix coordinators, TB health visitors, and senior treatment supervisors, need to be built to deal with the private sector more efficiently. Communication platforms and dialogue structures between sectors need to be strengthened to build mutual trust. NTEP may reconsider the amount of individual patient details required from the private sector and simplify mechanisms for data exchange between sectors. NTEP also needs to promote more nonfinancial incentives to the private sector, such as involving them in planning and review, recognizing and appreciating their efforts, and considering them as equal partners in the fight against TB. NTEP needs to strengthen policy to protect patient confidentiality and devise strategies to gain the confidence of citizens and private providers regarding the same. Private providers require periodic training on program components, such as Ni-kshay and Standards of TB Care. Customized electronic learning courses with flexible schedules and provisions for periodically updating the knowledge of private practitioners through an established communication channel could be attempted.

It is recommended to have a clear strategy for private-sector engagement that will help to avoid confusion among stakeholders.

### Strengths and Limitations

There are several strengths to this synthesis. The robust screening process ensured that only studies that addressed private-sector engagement for TB care with rich qualitative data were included. Qualitative assessment of the included studies offers transparency to the readers on the strengths and limitations of the studies included in this synthesis and consequently on any risk of bias in the interpretations presented and in conclusions drawn. We used a scientific approach to assess our confidence in each finding. However, there are some limitations, too. We may have missed some studies on the informal sector or chemists, as our search string was not sensitive enough to select those. Also, the synthesis includes only peer-reviewed publications in selected databases, therefore possibly losing out on some additional insights offered by other literature.

Another major limitation is the skewed geographical range of the studies included in the synthesis. Only about 30% of the studies included were from north India where private sector TB care, particularly the informal private sector, is far more prevalent than in south India. More studies regarding the issue are needed from the states of Uttar Pradesh, Maharashtra, Bihar, and Rajasthan, where the private sector is huge. As the number of eligible studies was very few, we did not conduct any subgroup analysis or interrogate the data to explore the impact of different types of providers, settings, or geographical areas on the barriers and facilitators to private-sector engagement. A few studies included were at least a decade old, and the context has changed a lot over the years, making the interpretations challenging.

Most of the studies included in the review were done at the district or subdistrict level, so country-level or policy-level challenges are not well documented. The Patient Provider Support Agency is a major strategy used by NTEP to engage the private sector; however, there are no published qualitative studies focusing on it. There are some interesting findings that might have huge policy implications, such as the use of schedule H1 regulation for surveillance, financial incentives for private providers, and the involvement of hospital administration. However, the evidence is of low quality as only a very few studies explored such topics. Studies focusing on the perspectives of chemists, informal health care providers, laboratory, AYUSH providers, and people affected with TB were only a few. Further studies could focus on all the above aspects. Better reporting is needed in qualitative research on this topic, particularly around recruitment methods, data collection methods, and the relationship between researcher and participants. There is also a need to use rigorous data analysis methods in such studies to increase confidence in the findings of qualitative studies.

## CONCLUSION

Factors related to the context in which the engagement occurs, the architecture of the engagement, and interaction among the actors contribute to barriers in engaging the private sector for TB care in India. Strengthening policies to protect patient confidentiality, using behavior change communication for NTEP program managers, providing managerial and soft-skill training to NTEP staff, promoting nonfinancial incentives to private providers, establishing a coordination mechanism between the sectors, and simplifying the data exchange mechanisms need to be done to further strengthen private-sector engagement.

## Notes

**Peer Reviewed**

**First Published Online:** July 18, 2024.
